# Tracheostomy in Flap‐Based Head and Neck Cancer Surgery: A Meta‐Analysis of Indications and Adverse Outcomes

**DOI:** 10.1002/hed.70102

**Published:** 2025-11-21

**Authors:** Raisa Chowdhury, Khanh Linh Tran, Naser Karimi, Jhorrit Kahlon, Cornelius Kürten, Sena Turkdogan, Eitan Prisman

**Affiliations:** ^1^ Faculty of Medicine and Health Sciences McGill University Montreal Quebec Canada; ^2^ Division of Otolaryngology, Department of Surgery University of British Columbia Vancouver British Columbia Canada; ^3^ Division of Otolaryngology – Head and Neck Surgery Vancouver General Hospital Vancouver British Columbia Canada; ^4^ Department of Otolaryngology‐Head and Neck Surgery McGill University Montreal Quebec Canada; ^5^ Royal College of Surgeons in Ireland Dublin Ireland

**Keywords:** airway, flaps, head and neck, operative, oral cancer, tracheostomy

## Abstract

**Background:**

Tracheostomy is frequently performed during flap‐based reconstruction for head and neck cancer, but predictive factors and complications are not well established.

**Methods:**

A systematic review and meta‐analysis was conducted per PRISMA guidelines. Studies of adult patients undergoing free or pedicled flap reconstruction were included. Pooled tracheostomy rates, predictors, and complications were analyzed using random‐effects models. Heterogeneity was assessed with the I^2^ statistic.

**Results:**

Twenty‐six studies (27 029 patients) were included. The pooled tracheostomy rate was 54.6%, decreasing to 42.4% when routine tracheostomy studies were excluded. Advanced tumor stage, oropharyngeal site, bilateral neck dissection, prior radiotherapy, and smoking predicted tracheostomy. Flap type was not significantly associated. The overall complication rate was 16.3%, including airway issues (2.6%). No significant change in tracheostomy rates was observed over 30 years.

**Conclusions:**

Tracheostomy use is influenced by tumor, surgical, and patient factors. Selective tracheostomy and validated risk tools may improve outcomes. Further prospective studies are needed.

## 1 Introduction

Head and neck cancers frequently necessitate extensive surgical resections followed by microvascular or pedicled flap‐based reconstructions to restore form and function [1]. Tracheostomy, whether temporary or permanent, is conventionally employed to secure the airway, prevent postoperative airway compromise, and facilitate pulmonary toilet, especially in patients at heightened risk due to bulky reconstructions, anticipated edema, or pre‐existing comorbidities [2]. The traditional principles held that tracheostomy should be routinely performed in all major flap‐based head and neck procedures to preempt devastating complications such as airway obstruction, aspiration, and emergent airway loss [3]. However, this approach is not without substantial risk, as tracheostomy itself can lead to a spectrum of complications including bleeding, infection, tracheal or stomal stenosis, pneumonia, tube dislodgement, and increased morbidity and resource utilization [2].

With the evolution of surgical techniques, perioperative protocols, and interdisciplinary care, an increasing number of centers are now challenging the necessity of universal tracheostomy in flap‐based head and neck surgeries. Recent studies have demonstrated that carefully selected patients may be safely managed without tracheostomy, reducing the risk of tracheostomy‐related complications and expediting recovery without compromising airway safety [4, 5]. Nevertheless, determining the optimal approach requires a nuanced understanding of patient‐ and procedure‐specific factors that predict the need for tracheostomy, balanced against the real risks of both airway compromise and procedure‐related sequelae.

Predictive factors such as anatomical site and extent of resection, type and size of reconstructive flap, anticipated postoperative swelling, comorbidity burden, prior radiation, and demographic factors such as age and body habitus [2]. Furthermore, the lack of universally accepted criteria has led to substantial practice variation, with decisions often reliant on surgeon experience rather than standardized risk assessments. A number of scoring systems have been proposed to stratify risk and standardize the decision‐making process, though their adoption remains limited across diverse clinical settings [6].

Complications following tracheostomy are significant, with studies reporting rates of bleeding, infection, decannulation, tracheal injury, and respiratory complications including pneumonia and acute respiratory distress syndrome [2]. Additionally, the prolonged presence of a tracheostomy tube can impact quality of life, prolong hospital stay, and increase healthcare costs. Importantly, recent evidence also underscores temporal shifts in tracheostomy practice patterns reflecting both improved perioperative care and evolving philosophies regarding risk reduction and patient‐centered outcomes. For instance, national trends indicate a fluctuating frequency of tracheostomy over time, with a shift toward earlier interventions and attempts to minimize duration and associated complications [7].

Therefore, there remains a pressing need for a comprehensive synthesis of contemporary evidence regarding the predictive factors, complication profiles, and temporal patterns of tracheostomy use in head and neck flap‐based surgeries. This systematic review and meta‐analysis aims to critically evaluate the current data, identify strong predictors for tracheostomy requirement, outline the incidence and nature of complications, and chart the evolution of clinical practice across recent decades.

## 2 Material and Methods

This study was conducted according to the Preferred Reporting Items for Systematic Reviews and Meta‐Analysis guidelines [8]. The study protocol was registered on PROSPERO under the ID CRD42023446325 [9].

### 2.1 Search Strategy

The search strategy was conducted using PubMed, Cochrane, Embase, Web of Science, and ClinicalTrials.gov databases. Articles were searched using medical subject headings (MeSH) terms: “tracheostomy”, “surgical procedures”, “operative”, “airway management”, “head and neck neoplasms”, “head and neck flap‐based surgeries”, “risk factors”, “patient selection”, “postoperative complications”, “demography”, “treatment outcome”, “time factors”, “length of stay”, “free flap surgery”, “surgical flaps”, “tracheotomy”, “surgical techniques”, “invasive procedures”, “non‐invasive procedures”, “comparative effectiveness research”. Exploded terms, shorthand forms and spelling variants were used as well as the Boolean operator AND between search terms for a more focused search. Reference lists in the selected articles were also searched for additional articles (Table S1).

### 2.2 Selection Criteria

Studies were included if they met the following criteria: (i) included adults (≥ 18 years) who underwent head and neck flap‐based surgeries (both free flaps and regional flaps); (ii) reported tracheostomy‐related outcomes such as predictive factors, postoperative complications, airway management, or surgical considerations; (iii) used study designs including retrospective or prospective cohort studies, case–control studies, cross‐sectional studies, longitudinal studies, case series, or clinical trials; and (iv) were published in English.

Studies were excluded if they: (i) were review articles, qualitative studies, textbook chapters, conference abstracts, commentaries, case reports, expert opinions, or editorials; (ii) lacked original data or a specific focus on tracheostomy outcomes in the context of head and neck flap‐based surgery; or (iii) provided incomplete or insufficient data relevant to the outcomes of interest. Two reviewers (RC and JK) independently screened titles, abstracts, and full texts using Covidence software [10]. Discrepancies were resolved through discussion and consensus.

### 2.3 Data Extraction

Two independent reviewers (RC and JK) extracted data from each included study, with disagreements resolved through discussion with a third reviewer (ST). Extracted information included study design, number of patients, demographics, tumor site and stage, flap type, neck dissection, prior treatments, and tracheostomy‐related outcomes.

The primary outcome was the identification of predictive factors for tracheostomy in head and neck flap‐based surgeries. Secondary outcomes included tracheostomy rates, complication types and rates, and relevant surgical or perioperative variables.

### 2.4 Assessment of Risk of Bias

Two authors (RC, JK) independently evaluated the quality of the studies using the ‘Cochrane Risk of Bias’ tool [11]. Based on the majority opinion, the risk of bias was assigned to each study. A study was considered a high‐quality study if it had a low risk of bias in most criteria assessed (Table S2).

### 2.5 Statistical Analysis

Meta‐analyses were performed using JBI SUMARI, applying a random‐effects model to pool tracheostomy rates and complication rates across studies, with heterogeneity assessed via the *I* ^2^ statistic [12, 13]. Proportions and odds ratios were synthesized for primary and secondary outcomes, and narrative synthesis was used where data were insufficient for pooling. Temporal trends were evaluated in MedCalc by plotting tracheostomy rates against study year and performing linear regression to assess for significant changes over time [14].

## 3 Results

### 3.1 Summary of Literature Search

**FIGURE 1:**
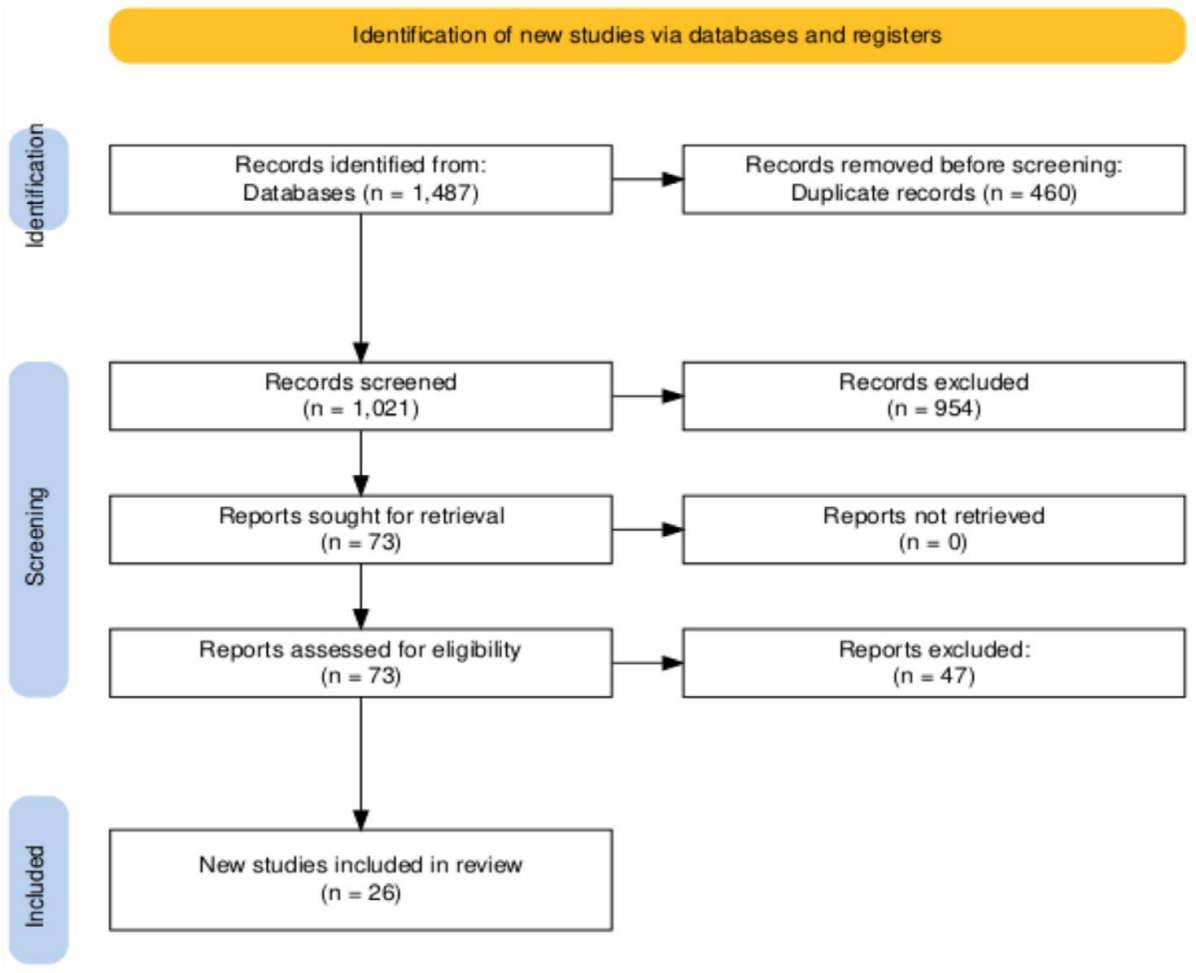
PRISMA flow chart. [Color figure can be viewed at wileyonlinelibrary.com]

The systematic literature search identified 1487 records from databases. After the removal of 460 duplicate records, 1021 articles remained and were screened by title and abstract. Of these, 73 articles were retrieved for full‐text assessment of eligibility. Following a detailed review, 47 articles were excluded based on predefined criteria. Ultimately, 26 studies were included in the final review [15, 16, 17, 18, 19, 20, 21, 22, 23, 24, 25, 26, 27, 28, 29, 30, 31, 32, 33, 34, 35, 36, 37, 38, 39, 40] (Figure 1) [41].

### 3.2 Study Characteristics

The systematic review included 26 studies published between 1994 and 2025, comprising entirely observational study designs. These consisted of 24 retrospective cohort studies, 1 combined retrospective and prospective cohort study and 1 purely observational study. No randomized controlled trials were identified. The studies were geographically diverse, with representation from Asia (China, India, Korea, Israel, Taiwan, and Pakistan), Europe (UK, Ireland, Germany, and Spain), North America (USA), and Australia.

Study sample sizes ranged from 20 to 18 416 participants. The mean or median age of participants generally ranged from 46 to 86 years, with most studies indicating a predominance of male patients. Several studies stratified outcomes based on surgical technique, anatomical site of the primary tumor, or type of flap used. Follow‐up durations and long‐term outcomes were inconsistently reported, though several studies included perioperative or short‐term postoperative complication rates (Table 1).

**TABLE 1:** Study characteristics.

Study and year	Design	Country	Sample size	Tracheostomy	Age (years)	Sex (M:F)
Adhikari et al. [17]	Retrospective	Australia	103	103	Median age: 64.6	72:31
Cai et al. [16]	Retrospective	China	664	390	Mean: 54.54 ± 14.9	329:204
Castling et al. [18]	Retrospective	USA	60	60	N/A	N/A
Chen et al. [19]	Retrospective	China	24	16	Median: 86 (range 85–91)	15:9
Davis et al. [20]	Retrospective	USA	50	2/24	83.7 (range 80–95)	33:17
Esteller et al. [21]	Retrospective	Spain	491	491	58.8 years (range 18.7–92.5 years)	436:55
Gupta et al. [22]	Retrospective	India	486	67	46.7 years, range 20 to 89	306:180
Halfpenny and McGurk [23]	Retrospective and prospective	UK	830	256	Median: 61 years (range 10–87)	178:87
Kruse‐Lösler et al. [24]	Retrospective	Germany	152	38	Mean: 58 years, range 32–84	N/A
Lee et al. [25]	Retrospective	Korea	24	12	Mean: PDT group: 66.3 ± 7.9, ST group: 68.14 ± 8.3	PDT group: 11:1, ST group: 10:2
Leiser et al. [15]	Retrospective review	Israel	75	27	60.9 (range 19–86)	50:25
Madgar et al. [26]	Retrospective review	Israel	109	68	Average was 59.7	53:56
Malata et al. [27]	Retrospective review	UK	89	89	Median 61 (17–79)	64:25
McDevitt et al. [28]	Observational study	Ireland	1651	428	61.1 years (range 20–97 years)	1205:1221
Meier et al. [29]	Retrospective observational study	Germany	47	25	62 years (range 38–85 years)	33:14
Mohamedbhai et al. [30]	Retrospective analysis of their own patients and a systematic review	N/A	149	82	N/A	N/A
Nagarkar et al. [31]	Retrospective review	India	500	13	Mean 67.15 (26–82)	N/A
Siddiqui et al. [32]	Retrospective review	Pakistan	400	81	N/A	289:111
Tassone et al. [33]	Retrospective review	USA	51	15	61.3 (40–85)	75:14
Xu et al. [34]	Retrospective review	China	1551	795	< 50 year = 533. > 50 year = 1018. (18–75)	1025:525
Holcomb et al. [35]	Retrospective cohort	USA	193	73		NA
Gupta et al. [37]	Retrospective study	India	66	6	Mean—50.3 years	54:12
Abdelrahman et al. [36]	Comparative observational study	UK	112	112	Percutaneous group mean—59.7, surgical tracheostomy group—61.9	
Kim et al. [39]	Retrospective cohort	Korea	20	10	Age‐mean—58.8 years in tracheostomy group	7;3
Cramer et al. [40]	Retrospective cohort	USA	861	551	61.4 ± 12.7	63%
Fang et al. [38]	Retrospective cohort	Taiwan	18 416	7981	55.2 ± 10.57	93.4%:6.6%

Of the 26 included studies, 9 explicitly evaluated tracheostomy‐related outcomes as their primary objective, while the remaining 17 reported tracheostomy data within larger cohorts assessing overall flap or reconstructive outcomes. In these latter studies, patients with and without tracheostomy were analyzed as distinct subgroups.

### 3.3 Summary of Quality Assessment

Quality assessment using the Newcastle–Ottawa Scale (NOS) [42] was conducted for all 26 included studies to evaluate the risk of bias across three domains: selection of participants (maximum 4 points), comparability of study groups (maximum 2 points), and outcome ascertainment (maximum 3 points). Of the 26 studies, 14 were rated as having a low risk of bias (total score ≥ 7), 10 studies demonstrated moderate risk of bias (score 5–6), and 2 studies were categorized as high risk (score < 5).

### 3.4 Tracheostomy Rates

**FIGURE 2:**
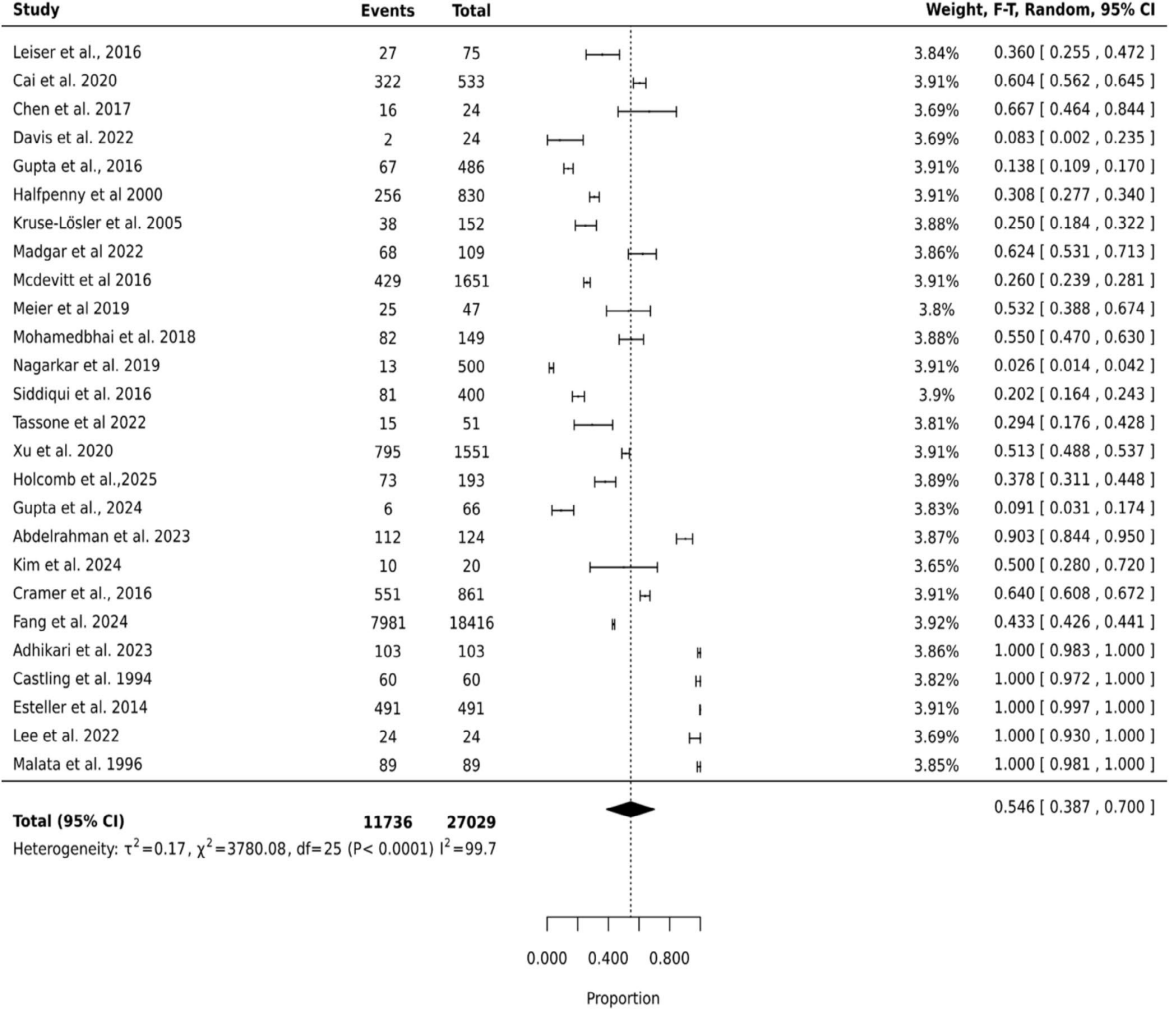
Forest plot overall tracheostomy rate.

A meta‐analysis of 26 studies with a cumulative sample size of 27 029 patients undergoing head and neck flap‐based surgery found that the pooled tracheostomy rate was 54.6% (95% CI: 38.7%–70.0%). Heterogeneity among the studies was extremely high at *I* ^2^ = 99.7%, *p* < 0.0001, *τ* ^2^ = 0.17 (Figure 2).

#### 3.4.1 Sensitivity Analysis of Tracheostomy Rate

A sensitivity analysis was conducted to assess the strength of the pooled tracheostomy rate by excluding studies that reported a 100% tracheostomy rate. After removal of these outlier studies, the meta‐analysis of 22 studies comprising 26 753 patients yielded a pooled tracheostomy rate of 42.4% (95% CI: 29.6%–55.8%). Heterogeneity remained extremely high (*I* ^2^ = 99.6%, *p* < 0.0001, *τ* ^2^ = 0.10), indicating persistent variability in reported rates across the included studies (Figure S1).

### 3.5 Primary Outcomes

#### 3.5.1 Predictive Factors

##### 3.5.1.1 T3–T4 Versus T1–T2

**FIGURE 3:**
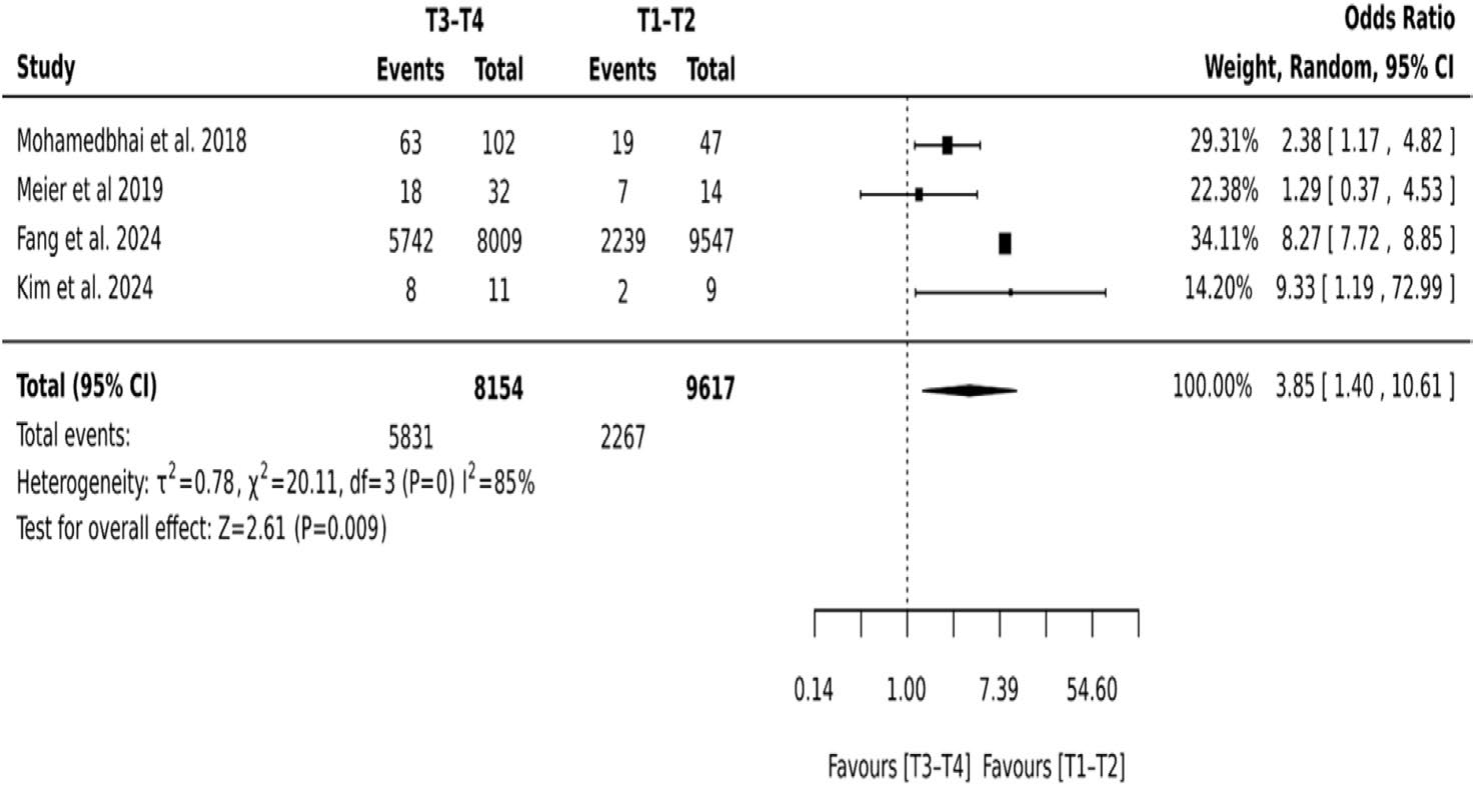
Forest plot–T4–T3 versus T2–T1.

Across four studies encompassing 17 771 patients, tumor stage demonstrated a strong association with the likelihood of tracheostomy. Patients presenting with advanced‐stage tumors (T3–T4) were significantly more likely to undergo tracheostomy compared to those with early‐stage disease (T1–T2), with a pooled odds ratio of 3.85 (95% CI: 1.40–10.61; *p* = 0.009). There was considerable heterogeneity observed across the studies (*I* ^2^ = 85%) (Figure 3).

##### 3.5.1.2 Oropharynx Versus Oral Cavity

**FIGURE 4:**
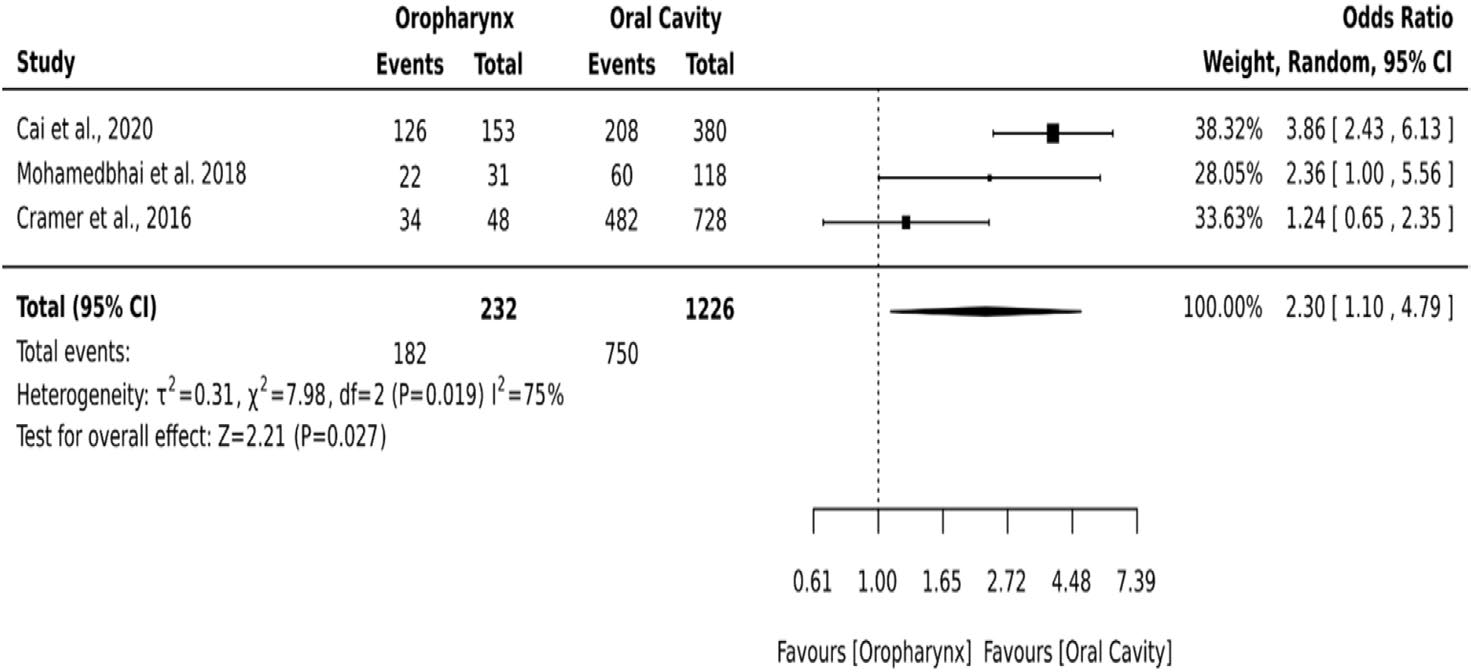
Forest plot oropharynx versus oral cavity.

A subgroup meta‐analysis of three studies (*n* = 1458) comparing oropharyngeal and oral cavity (anterior two‐thirds of the tongue (mobile tongue), floor of mouth, buccal mucosa, lower and upper gingivae (alveolar ridges), retromolar trigone, hard palate, and the mucosal surfaces of the lips) tumor sites revealed a significantly higher likelihood of tracheostomy in patients with oropharyngeal malignancies (OR = 2.30; 95% CI: 1.10–4.79; *p* = 0.027). However, the analysis showed substantial heterogeneity (*I* ^2^ = 75%), indicating variability in study populations or perioperative practices (Figure 4).

##### 3.5.1.3 Bilateral Versus Unilateral Neck Dissection

**FIGURE 5:**
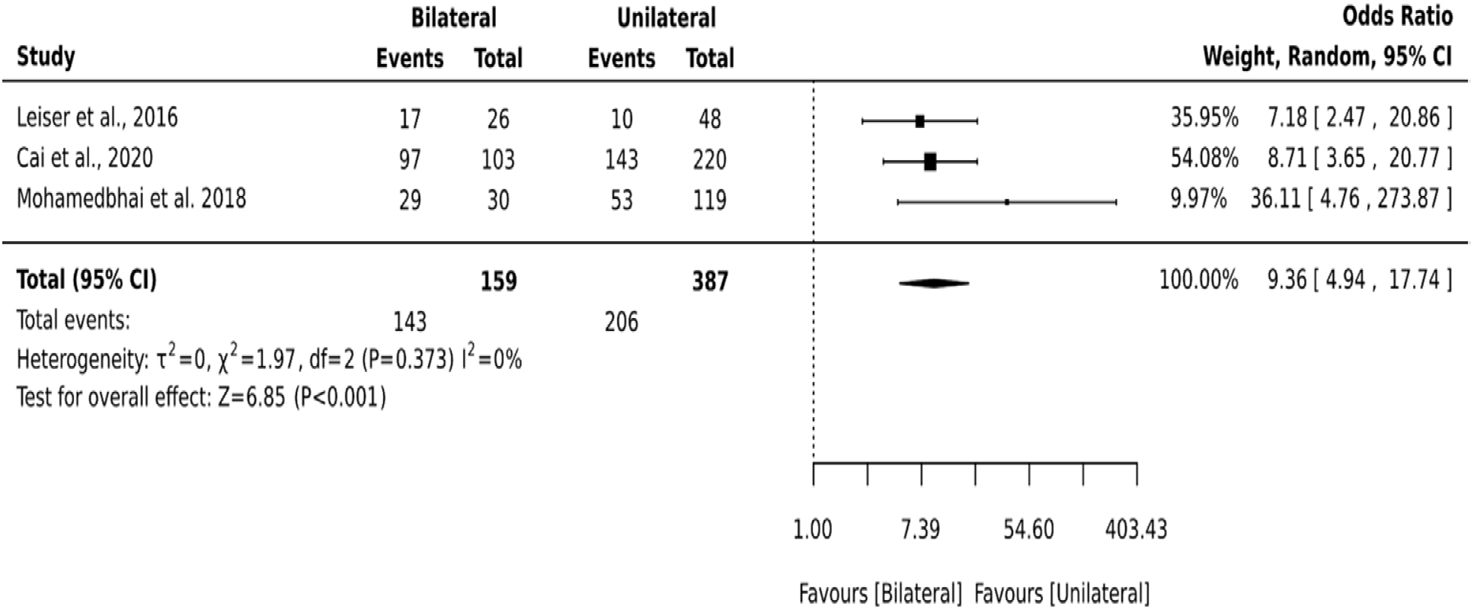
Forest plots—bilateral versus unilateral neck dissection.

Bilateral neck dissection emerged as a strong predictive factor for tracheostomy in head and neck flap‐based cancer surgery. A subgroup meta‐analysis of three studies (*n* = 546) comparing bilateral and unilateral neck dissection demonstrated a markedly higher risk of tracheostomy among patients undergoing bilateral procedures (OR = 9.36; 95% CI: 4.94–17.74; *p* < 0.001). Notably, the analysis found no significant heterogeneity across studies (*I* ^2^ = 0%) (Figure 5).

##### 3.5.1.4 Free Flap Versus Pedicled

The incidence of tracheostomy did not significantly differ between patients undergoing free flap versus pedicled flap reconstruction in head and neck cancer surgery (OR, 1.95 [95% CI, 0.57–6.68]; *I* ^2^ = 75%; 262 patients). Substantial heterogeneity was observed (*I* ^2^ = 75%) (Figure S2).

##### 3.5.1.5 Patient‐Related Predictive Factors

Smoking was consistently shown to increase the risk of tracheostomy: Leiser et al. [15] reported that heavy smokers had significantly increased odds (OR 6.77, 95% CI 1.79–25.54, *p* = 0.002) a finding corresponding with a study by Cai et al. [16], who also observed a higher risk among smokers (OR 2.6, *p* = 0.004). McDevitt et al. [28], Cramer et al. [40], and Meier et al. [29], also observed higher rates of tracheostomy among patients with any smoking history, underlining the role of compromised airway and tissue health in this group.

Prior radiotherapy was similarly found to be a strong predictive factor. Cai et al. [16] found previous radiotherapy associated with over fourfold increased odds of requiring tracheostomy (OR 4.11, *p* = 0.0032), and findings supported by Xu et al. [34] (OR 3.37, 95% CI 1.87–6.09, *p* < 0.001) and Mohamedbhai et al. [30] (*p* < 0.0001).

Comorbidities were variably reported. Leiser et al. [15] highlighted that all tracheostomized patients had major medical comorbidities, yet larger and more detailed cohorts, such as Cai et al. [16], found no significant associations for diabetes (OR 1.56, *p* = 0.11) or hypertension (OR 1.01, *p* = 0.99) as isolated factors. However, Kruse‐Lösler et al. [24] observed a significant increase in tracheostomy risk among patients with three or more chronic diseases (*p* < 0.05). Due to wide variability in study design and reporting, a formal pooled meta‐analysis was not feasible for these patient‐related factors (Table 2).

**TABLE 2:** Predictive factors.

Study	Factor	Sub‐factor	Tracheostomy (*n*)	Total
Fang et al. [38]	Tumor stage	T1–T2	2239	9547
		T3–T4	5742	8009
Cramer et al. [40]	Tumor site	Oral cavity	482	728
		OroPharynx	34	48
Kim et al. [39]	Tumor site	Oral cavity	9	19
		OroPharynx	1	1
	Tumor stage	T1–T2	2	9
		T3–T4	8	11
Mohamedbhai et al. [30]	Tumor stage	T1–T2	19	47
		T3–T4	63	102
	Tumor site	Oral cavity	60	118
		OroPharynx	22	31
	Neck dissection	Bilateral	29	30
		Unilateral	53	119
Meier et al. [29]	Tumor stage	T1–T2	7	14
		T3–T4	18	32
	Flap type	Microvascular (mvf) free	19	34
		Pedicled regional	6	13
Kruse‐Lösler et al. [24]	Flap type	Radial forearm flap (free)	26	59
		Local flap (pedicled)	12	93
Madgar et al. [26]	Neck dissection	Bilateral	14	17
		Unilateral	39	60
Leiser et al. [15]	Neck dissection	Bilateral	17	46
		Unilateral	10	48
	Flap type	Pectoralis major (pedicled)	7	18
		Radial forearm (free)	15	45
Cai et al. [16]	Neck dissection	Bilateral	97	103
		Unilateral	143	220
	Tumor site	Oral cavity	208	380
		OroPharynx	126	153

#### 3.5.2 Complications

##### 3.5.2.1 Overall Complications

Six studies (825 patients) reported on overall complication rates. Significant heterogeneity was observed across studies (*I* ^2^ = 93.2%, *p* < 0.0001), and the pooled complication rate was 16.3% (95% CI: 6.9%–28.5%) using a random effects model. Individual study rates varied considerably, ranging from 7.4% to 43.8%, reflecting substantial differences in reporting practices, case mix, and perioperative protocols (Table 3) (Figure S3).

**TABLE 3:** Complications.

Study	Complication type	*N* (tracheostomy)	*N* (with complication)
Cai et al. [16]	Any tracheostomy complication	322	27
	Pneumonia	322	14
	Hemorrhage	322	12
	Subcutaneous emphysema	322	4
	Accidental dislodgement	322	2
	Multiple complications	322	5
Adhikari et al. [17]	Any complication (overall)	103	37
Leiser et al. [15]	Infected dehiscence	27	6
Castling et al. [18]	Any complication (overall)	60	7
	Displaced tube	60	1
	Chest infection	60	5
	Obstructed tube	60	1
Chen et al. [19]	Overall	16	7
Esteller et al. [21]	Stomal recurrence	491	6
	Death after recurrence	6	5
	Disease control after salvage	3	1
Halfpenny and McGurk [23]	Overall	256	19
Madgar et al. [26]	Any trach‐related complication	68	7
	Tracheal stenosis	68	1
	Supra‐stomal stenosis	68	2
	Stomal bleeding	68	2
	Stomal infection	68	1
	Pneumonia	68	4
Malata et al. [27]	Chest infection	89	15
	Surgical emphysema	89	1
	Minor stomal bleeding	89	1
	Bilateral pleural effusion	89	1
Abdelrahman et al. [36]	Tube obstruction	112	3
Cramer et al. [40]	Pneumonia	551	49
	SSI	551	76
	Wound dehiscence	551	32
	Sepsis	551	31
	Death	551	3
	Bleeding requiring transfusion	551	203
	Airway‐specific complications	551	46
Meier et al. [29]	Pneumonia	25	1
	Delirium	25	2

##### 3.5.2.2 Chest Infections

A meta‐analysis of six studies (*n* = 1115) evaluated the rate of chest infections principally postoperative pneumonia and other chest infections following head and neck flap‐based surgeries. The pooled proportion for chest infection was 7.7% (95% CI: 4.4%–11.6%), determined using a random effects model. Significant heterogeneity was observed (*I* ^2^ = 69.3%, *p* = 0.0095) (Figure S4).

##### 3.5.2.3 Other Infections

An analysis of five studies (*n* = 1748 patients) assessed other infection‐related complications, including surgical site infection (SSI), septic shock, stomal infection, infected dehiscence and wound dehiscence. The pooled overall infection complication rate was 7.8% (95% CI: 3.0%–14.3%), with significant heterogeneity (*I* ^2^ = 93.3%, *p* < 0.0001) (Figure S5).

##### 3.5.2.4 Tube‐Related Complications

Tube‐related complications consisting of studies with accidental dislodgement, obstructed tube, and displaced tube were analyzed across five studies (*n* = 614) following tracheostomy in head and neck surgery patients. The pooled overall rate was 1.2% (95% CI: 0.002–0.026). Heterogeneity across studies was low (*I* ^2^ = 21.8%, *p* = 0.455).

##### 3.5.2.5 Airway‐Related Complications

Meta‐analysis of airway‐related complications across five studies (*n* = 1098), covering tracheal stenosis, supra‐stomal stenosis, and surgical/subcutaneous emphysema had a pooled rate of 2.6% (95% CI: 0.005–0.057). Moderate heterogeneity was observed (*I* ^2^ = 80%, *p* < 0.0001).

##### 3.5.2.6 Bleeding

Bleeding complications had a pooled incidence of 8.1% (95% CI: 0.0%–25.9%). However, heterogeneity was substantial (*I* ^2^ = 98%, *p* < 0.0001), which may reflect broad differences in how bleeding was defined, patient risk factors, or surgical technique.

#### 3.5.3 Temporal Trends

A linear regression analysis of tracheostomy rates over time across studies revealed no significant temporal trend, with the regression equation *y* = −199.627 + 0.121*x*, a correlation coefficient of *r* = 0.06 and a non‐significant *p*‐value (*p* = 0.783).

#### 3.5.4 Publication Bias

Assessment of publication bias was not formally performed using funnel plots with Egger's test for most outcomes, as these methods are not appropriate when the number of studies is limited. According to established methodological guidance, funnel plots generated from less than 10 studies lack the statistical power to distinguish between true asymmetry and random variation. The marked heterogeneity in study design, effect measures, and outcome reporting observed across our meta‐analyses could contribute to visually misleading funnel plots, increasing the risk of subjective misinterpretation. Tracheostomy rates and complication outcomes in this review were based on pooled proportions, rather than comparative effect sizes, making traditional funnel plot assessment inappropriate for these endpoints [43].

## 4 Discussion

This meta‐analysis of 26 studies, encompassing over 27 029 patients undergoing head and neck flap‐based surgeries, found that tracheostomy remains a common intervention, with a pooled rate of 54.6%. On excluding studies that routinely performed tracheostomy in all cases, the rate still remained substantial at 42.4%, indicating that a significant proportion of patients undergoing these complex surgeries require airway protection Patients with advanced tumors (T3–T4) were significantly more likely to undergo tracheostomy than those with early‐stage disease (OR = 3.85; 95% CI: 1.40–10.61; *p* = 0.009). Tumor location also played an important role, with oropharyngeal tumors demonstrating higher odds of tracheostomy compared to oral cavity tumors (OR = 2.30; 95% CI: 1.10–4.79; *p* = 0.027). Bilateral neck dissection emerged as the most powerful procedural predictor, associated with nearly a 9.4‐fold increase in tracheostomy risk (OR = 9.36; 95% CI: 4.94–17.74; *p* < 0.001). Patient‐related factors such as prior radiotherapy and smoking were also strongly associated. Interestingly, the type of flap used—free versus pedicled—did not significantly influence tracheostomy risk (OR = 1.95; 95% CI: 0.57–6.68). The pooled complication rate following tracheostomy was 16.3%, with common adverse events including chest infections (7.7%), surgical site or wound‐related infections (7.8%), bleeding (8.1%), and airway complications such as stenosis or dislodgement (2.6%). Notably, no meaningful temporal trend in tracheostomy rates was observed over the past three decades (*p* = 0.783), suggesting persistent variability in practice despite evolving surgical standards.

The elevated risk of tracheostomy in patients with T3–T4 tumors reflects the increased airway compromise and surgical complexity associated with these cases. This corresponds with a study by Eissner et al. [44] that found patients with advanced tumors had a 5.7 times higher risk (OR 5.7; 95% CI: 1.48–21.9) for unplanned tracheostomy compared to those with early‐stage disease [44]. Similarly Gong et al. found that preoperative tracheostomy was more common in T4 tumors (36.8%) compared to T3 (12.2%), particularly in tumors exceeding 10 cm^2^ in size [45]. These patients often require multilevel surgery, longer operative time, and more bulky reconstructions all of which increase the risk of postoperative edema and airway obstruction.

Our findings showing elevated tracheostomy rates for oropharyngeal tumors are supported by Neal et al. [46], who noted that cancers involving the base of the tongue and supraglottic region were more likely to result in prolonged tracheostomy dependence. These areas lie in close proximity to the upper airway and are prone to substantial postoperative swelling, making elective tracheostomy a safer and often necessary option in these patients.

Bilateral neck dissection emerged as one of the strongest predictors for tracheostomy. Extensive dissection disrupts lymphatic drainage, increases the risk of soft tissue edema, and may reduce neck mobility, all of which compromise airway patency [47]. The absence of heterogeneity across studies (*I* ^2^ = 0%) strengthens the reliability of this association. These findings emphasize the need to consider surgical extent, rather than tumor factors alone, when planning perioperative airway management. It is important to note that bilateral neck dissection may partially reflect confounding by tumor site and stage, as more extensive oropharyngeal and bilateral oral cavity tumors frequently necessitate bilateral dissection. The limited granularity of reported data precluded statistical adjustment for this inter‐relationship.

In contrast, the lack of a significant association between flap type and tracheostomy suggests that reconstructive technique, in isolation, should not dictate airway strategy. As highlighted by Madgar et al. [26], flap bulk and inset location may be more relevant than donor site, reinforcing the importance of individualized assessment of flap volume and its impact on airway compression.

Radiotherapy was another consistent predictor. Pre‐irradiated tissues are less compliant, more prone to edema, and heal poorly, all of which increase the likelihood of airway compromise. Cai et al. [16], Xu et al. [34], and Mohamedbhai et al. [30] all found significantly higher odds of tracheostomy in previously irradiated patients. This aligns with the findings of Mijiti et al. [48], who showed that preoperative radiotherapy increased flap‐related complications and worsened outcomes [48].

Not all comorbidities—specifically hypertension and diabetes—were strong predictors. Cai et al. [16] reported no significant associations for these variables, though Kruse‐Lösler et al. [24] noted increased tracheostomy use in patients with three or more comorbidities, suggesting that cumulative health burden may be more informative than individual diagnoses.

Our findings also have important clinical implications for airway decision‐making. Several validated scoring systems have been developed to guide tracheostomy planning, including the Peking University score [16] and the TRACHY score [49]. These tools incorporate key risk factors identified in our analysis such as tumor stage, neck dissection, radiotherapy, and flap characteristics and offer structured, risk‐based thresholds for decision‐making. Integrating these tools into routine preoperative planning could reduce unnecessary tracheostomies while ensuring airway safety in high‐risk cases.

Our findings support risk‐adapted alternatives to routine tracheostomy, such as delayed extubation or nasotracheal intubation, which have shown promising results in select patients. Moore et al. reported successful outcomes with nasotracheal intubation in free flap patients, noting shorter hospital stays and reduced dependency on feeding tubes [50]. Similarly, Gigliotti et al. [51] found that nasotracheal intubation as part of an Enhanced Recovery After Surgery (ERAS) protocol improved ICU outcomes and accelerated recovery [51]. These strategies, when applied to low‐ or moderate‐risk patients, may reduce tracheostomy‐related complications and enhance overall recovery and warrant further exploration in future research. The study found a 16.3% overall complication rate related to tracheostomy. These risks are clinically significant, especially in frail or comorbid patients. Reported complication rates in our cohort are comparable to those described in general tracheostomy populations (14.2% in a recent meta‐analysis of adults), underscoring that flap‐based reconstruction itself does not appear to disproportionately increase tracheostomy morbidity [52]. Madgar et al. [26] reported greater morbidity in tracheostomized patients compared to those managed with delayed extubation. Long‐term complications such as tracheal stenosis, vocal impairment, and psychosocial burden are often underappreciated yet relevant in functional outcomes. Thus, risk‐adapted tracheostomy planning is not only a matter of safety but also of quality of life and resource stewardship.

While airway‐related complications such as tracheal or supra‐stomal stenosis were captured (pooled 2.6%), long‐term surveillance was inconsistently reported across studies. Consequently, delayed post‐decannulation stenosis may be under‐recognized, highlighting the need for standardized long‐term follow‐up in future research.

This study is not without limitations. High heterogeneity across studies reflects variability in institutional practices, patient populations, and surgical techniques. Most studies were retrospective, with moderate‐to‐high risk of bias, and several lacked complete data on event rates or confounders. Definitions for tracheostomy indications and complications also varied, limiting comparability. Furthermore, scoring systems while promising remain underutilized and lack widespread external validation.

## 5 Conclusion

This meta‐analysis confirms that advanced tumor stage, oropharyngeal location, bilateral neck dissection, prior radiotherapy, and smoking are significant predictors of tracheostomy in head and neck flap‐based surgery. Complication rates, though manageable, are non‐negligible. Risk‐based airway planning supported by validated scoring systems and enhanced recovery protocols can reduce unnecessary tracheostomies and improve patient outcomes. Future prospective studies should aim to validate predictive tools, standardize definitions, and evaluate patient‐centered outcomes, helping shape individualized, evidence‐based airway strategies in this complex surgical population.

## Funding

The authors have nothing to report.

## Ethics Statement

The authors have nothing to report.

## Conflicts of Interest

The authors declare no conflicts of interest.

## Supporting information


**FIGURE S1:** Forest plot bleeding rates.

**FIGURE S2:** Forest plots tracheal stenosis.

**FIGURE S3:** Forest plots accidental dislodgement.

**FIGURE S4:** Forest plot obstructed tracheostomy.

**FIGURE S5:** Forest plot infections.

**TABLE S1:** Search strategy.

**TABLE S2:** Newcastle‐Ottawa Scale (NOS) risk of bias assessment table for all included studies.

## Data Availability

Data sharing not applicable to this article as no datasets were generated or analysed during the current study.
