# Supplementary figures and images for: Tracheostomy in Flap‐Based Head and Neck Cancer Surgery: A Meta‐Analysis of Indications and Adverse Outcomes
Source: Head Neck. 2025 Nov 21;48(2):570–82. doi: 10.1002/hed.70102 (PMC12797017; doi:10.1002/hed.70102)

**Supplementary figure S1: Forest plot Bleeding rates**


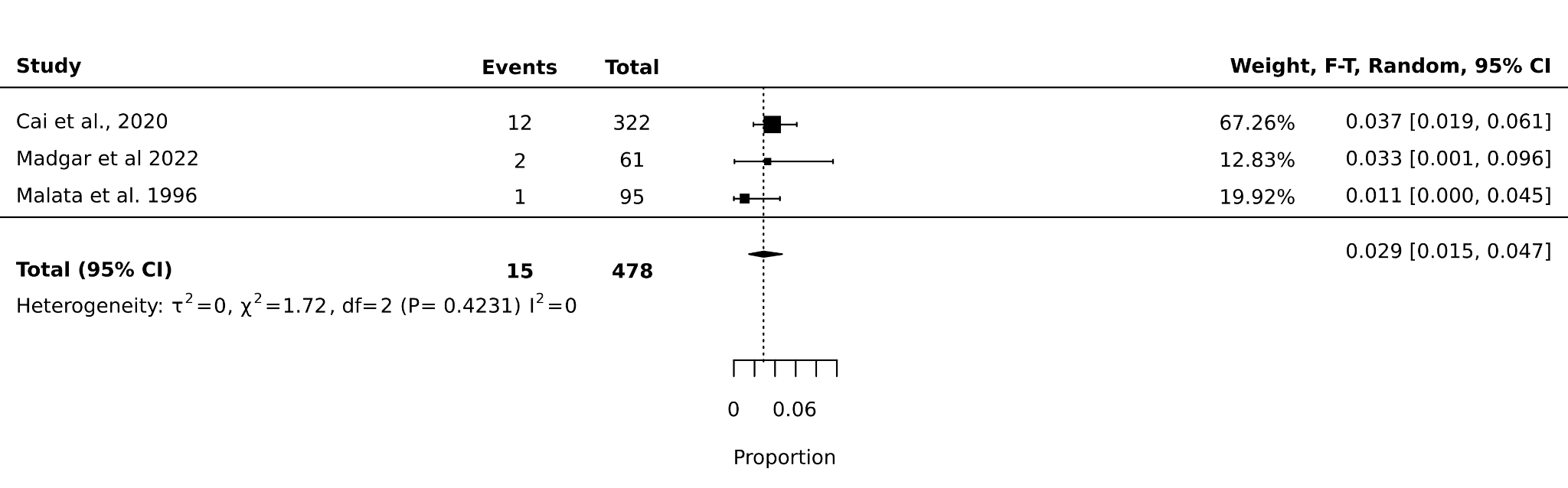

Supplement: Supplementary file 1 — FIGURE S1: Forest plot bleeding rates. [file HED-48-570-s003.docx]

**Supplementary Figure S2: Forest Plots Tracheal Stenosis**


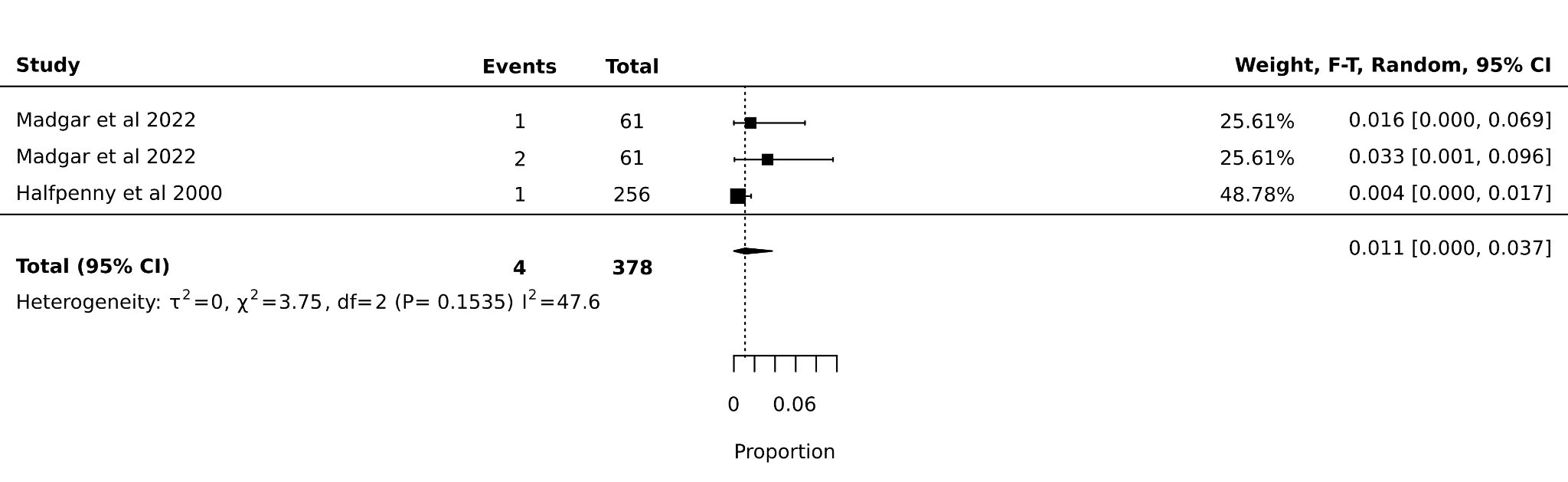

Supplement: Supplementary file 2 — FIGURE S2: Forest plots tracheal stenosis. [file HED-48-570-s001.docx]

**Supplementary Figure S3: Forest Plots Accidental dislodgement**


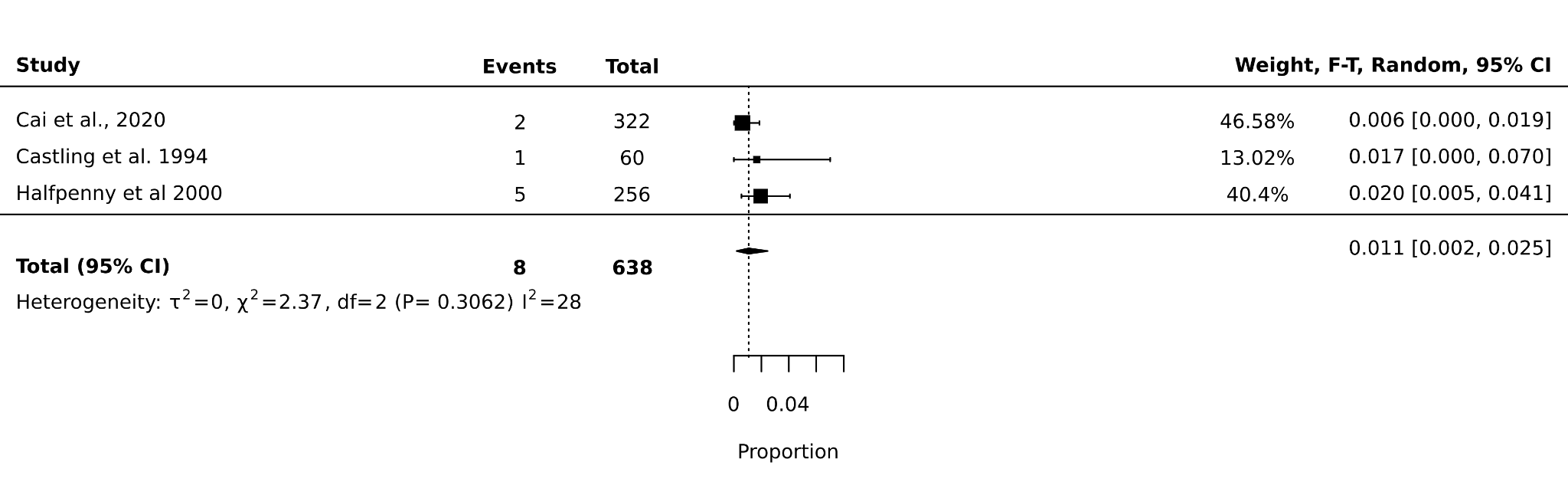

Supplement: Supplementary file 3 — FIGURE S3: Forest plots accidental dislodgement. [file HED-48-570-s002.docx]

**Supplementary Figure S4: Forest plot Obstructed Tracheostomy**


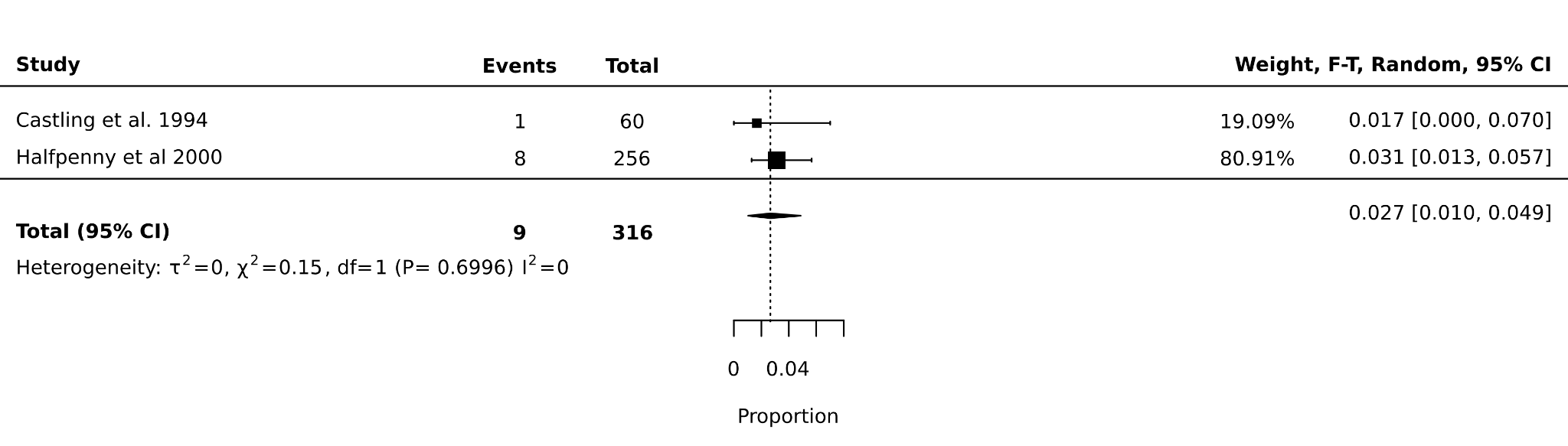

Supplement: Supplementary file 4 — FIGURE S4: Forest plot obstructed tracheostomy. [file HED-48-570-s005.docx]

**Supplementary Figure S5: Forest plot Infections**


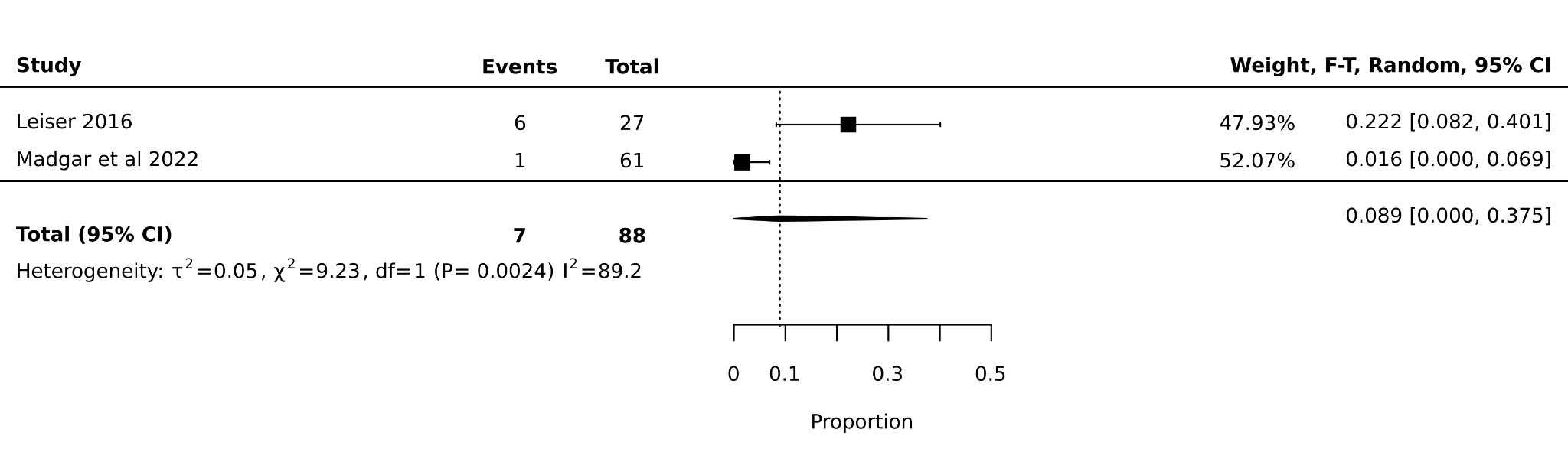

Supplement: Supplementary file 5 — FIGURE S5: Forest plot infections. [file HED-48-570-s004.docx]
