# Supplementary material for: Tracheostomy in Flap‐Based Head and Neck Cancer Surgery: A Meta‐Analysis of Indications and Adverse Outcomes
Source: Head Neck. 2025 Nov 21;48(2):570–82. doi: 10.1002/hed.70102 (PMC12797017; doi:10.1002/hed.70102)
Supplement: Supplementary file 6 — TABLE S1: Search strategy. [file HED-48-570-s007.docx]

**Supplementary Table 1: Search Strategy**

| Database | Search Terms Used |
| --- | --- |
| PubMed | ("tracheostomy" OR "tracheotomy" OR "airway management") AND ("head and neck neoplasms" OR "head and neck cancer" OR "head and neck surgeries") AND ("free flap surgery" OR "reconstructive surgery" OR "flap-based surgery") AND ("perioperative complications" OR "surgical outcomes" OR "airway obstruction") AND ("elective tracheostomy" OR "emergency tracheostomy" OR "prophylactic tracheostomy") |
| Cochrane | ("tracheostomy" OR "surgical airway" OR "perioperative airway management") AND ("head and neck cancer" OR "oral cancer" OR "laryngeal cancer") AND ("flap reconstruction" OR "free flap surgery" OR "surgical techniques") AND ("risk factors" OR "predictors" OR "prognostic factors") AND ("postoperative complications" OR "length of hospital stay" OR "decannulation timing") |
| Embase | ("tracheostomy" OR "tracheotomy" OR "elective airway management") AND ("head and neck malignancies" OR "oropharyngeal cancer" OR "laryngectomy") AND ("surgical flaps" OR "pedicled flaps" OR "free tissue transfer") AND ("hospital outcomes" OR "morbidity and mortality" OR "infection rates") AND ("comparative analysis" OR "effectiveness research" OR "tracheostomy utilization trends") |
| Web of Science | ("tracheostomy" OR "temporary airway" OR "postoperative airway management") AND ("head and neck tumor" OR "advanced T3 T4 cancer" OR "tumor staging") AND ("bilateral neck dissection" OR "reconstructive surgery" OR "airway risk assessment") AND ("airway compromise" OR "tracheostomy complications" OR "hospital readmission") AND ("historical trends" OR "temporal changes" OR "surgical evolution") |
| ClinicalTrials.gov | ("tracheostomy" OR "airway intervention" OR "tracheostomy necessity") AND ("head and neck flap-based reconstruction" OR "oral cavity malignancy" OR "surgical treatment") AND ("airway obstruction risk" OR "tumor size predictors" OR "surgical case complexity") AND ("patient selection criteria" OR "anesthesia considerations" OR "perioperative airway planning") AND ("systematic review" OR "meta-analysis" OR "retrospective analysis") |

**Supplementary Table 2: Newcastle-Ottawa Scale (NOS) Risk of Bias Assessment Table for all included studies**

| **Study Name** | **Selection (4 Points)** | **Comparability (2 Points)** | **Outcome (3 Points)** | **Total Score** | **Risk of Bias Level** |
| --- | --- | --- | --- | --- | --- |
| Adhikari *et al*. (2023) [15] | 4 | 2 | 3 | 9 | Low |
| Halfpenny & McGurk (2000) [21] | 4 | 1 | 3 | 8 | Low |
| Mohamedbhai *et al*. (2018) [28] | 4 | 2 | 2 | 8 | Low |
| Xu *et al.* (2021) [32] | 4 | 1 | 3 | 8 | Low |
| Nagarkar *et al.* (2019) [29] | 3 | 1 | 3 | 7 | Moderate |
| Esteller *et al.* (2014) [19] | 3 | 1 | 2 | 6 | Moderate |
| Leiser *et al.* (2016) [5] | 4 | 2 | 3 | 9 | Low |
| Siddiqui *et al.* (2016) [30] | 3 | 1 | 2 | 6 | Moderate |
| Malata *et al.* (1996) [25] | 4 | 2 | 3 | 9 | Low |
| Chen et al. (2017) [17] | 4 | 2 | 2 | 8 | Low |
| Madgar *et al.* (2022) [24] | 3 | 2 | 2 | 7 | Moderate |
| Kruse-Lösler *et al.* (2005) [22] | 4 | 1 | 2 | 7 | Moderate |
| Lee et al. (2022) [23] | 4 | 1 | 3 | 8 | Low |
| McDevitt *et al.* (2016) [26] | 3 | 2 | 3 | 8 | Low |
| Meier *et al.* (2023) [27] | 4 | 2 | 3 | 9 | Low |
| Gupta *et al*. (2016) [20] | 3 | 1 | 2 | 6 | Moderate |
| Cai *et al.* (2019) [7] | 4 | 1 | 3 | 8 | Low |
| Tassone *et al.* (2022) [31] | 3 | 2 | 3 | 8 | Low |
| Davis *et al.* (2022) [18] | 3 | 2 | 2 | 7 | Moderate |
