# Supplementary material for: Tracheostomy in Flap‐Based Head and Neck Cancer Surgery: A Meta‐Analysis of Indications and Adverse Outcomes
Source: Head Neck. 2025 Nov 21;48(2):570–82. doi: 10.1002/hed.70102 (PMC12797017; doi:10.1002/hed.70102)
Supplement: Supplementary file 7 — TABLE S2: Newcastle‐Ottawa Scale (NOS) risk of bias assessment table for all included studies. [file HED-48-570-s006.docx]

**Supplementary Table 2: Newcastle-Ottawa Scale (NOS) Risk of Bias Assessment Table for all included studies**

| **Study Name** | **Selection (4 Points)** | **Comparability (2 Points)** | **Outcome (3 Points)** | **Total Score** | **Risk of Bias Level** |
| --- | --- | --- | --- | --- | --- |
| Adhikari *et al*. (2023) [15] | 4 | 2 | 3 | 9 | Low |
| Halfpenny & McGurk (2000) [21] | 4 | 1 | 3 | 8 | Low |
| Mohamedbhai *et al*. (2018) [28] | 4 | 2 | 2 | 8 | Low |
| Xu *et al.* (2021) [32] | 4 | 1 | 3 | 8 | Low |
| Nagarkar *et al.* (2019) [29] | 3 | 1 | 3 | 7 | Moderate |
| Esteller *et al.* (2014) [19] | 3 | 1 | 2 | 6 | Moderate |
| Leiser *et al.* (2016) [5] | 4 | 2 | 3 | 9 | Low |
| Siddiqui *et al.* (2016) [30] | 3 | 1 | 2 | 6 | Moderate |
| Malata *et al.* (1996) [25] | 4 | 2 | 3 | 9 | Low |
| Chen et al. (2017) [17] | 4 | 2 | 2 | 8 | Low |
| Madgar *et al.* (2022) [24] | 3 | 2 | 2 | 7 | Moderate |
| Kruse-Lösler *et al.* (2005) [22] | 4 | 1 | 2 | 7 | Moderate |
| Lee et al. (2022) [23] | 4 | 1 | 3 | 8 | Low |
| McDevitt *et al.* (2016) [26] | 3 | 2 | 3 | 8 | Low |
| Meier *et al.* (2023) [27] | 4 | 2 | 3 | 9 | Low |
| Gupta *et al*. (2016) [20] | 3 | 1 | 2 | 6 | Moderate |
| Cai *et al.* (2019) [7] | 4 | 1 | 3 | 8 | Low |
| Tassone *et al.* (2022) [31] | 3 | 2 | 3 | 8 | Low |
| Davis *et al.* (2022) [18] | 3 | 2 | 2 | 7 | Moderate |
